# *N*,*N*′-Dimeth­oxy-*N*,*N*′-dimethyl­succinamide

**DOI:** 10.1107/S1600536808018369

**Published:** 2008-06-21

**Authors:** Sumei Yao

**Affiliations:** a Medical College of Henan University, Henan University, Kaifeng 475004, People’s Republic of China

## Abstract

The title compound, C_8_H_16_N_2_O_4_, is a Weinreb amide that is also an important inter­mediate for the preparation of ketones and aldehydes. The molecule possesses a centre of symmetry.

## Related literature

For related literature, see: Nahm & Weinreb (1981 ▶).
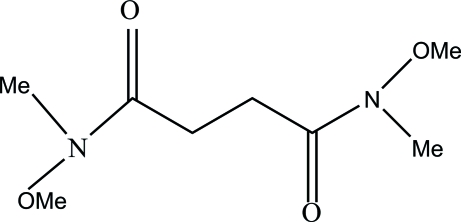


## Experimental

### 

#### Crystal data

C_8_H_16_N_2_O_4_
*M* *_r_* = 204.23
Monoclinic, 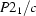

*a* = 4.2645 (15) Å
*b* = 11.152 (4) Å
*c* = 11.165 (4) Å
β = 98.485 (5)°
*V* = 525.2 (3) Å^3^
*Z* = 2
Mo *K*α radiation
μ = 0.10 mm^−1^
*T* = 296 (2) K
0.20 × 0.16 × 0.13 mm

#### Data collection

Bruker SMART APEX CCD area-detector diffractometer
Absorption correction: multi-scan (*SADABS*; Sheldrick, 2001 ▶) *T*_min_ = 0.980, *T*_max_ = 0.987
2116 measured reflections
909 independent reflections
776 reflections with *I* > 2σ(*I*)
*R*_int_ = 0.015

#### Refinement

*R*[*F* ^2^ > 2σ(*F* ^2^)] = 0.055
*wR*(*F* ^2^) = 0.174
*S* = 1.01
909 reflections
64 parameters
H-atom parameters constrained
Δρ_max_ = 0.25 e Å^−3^
Δρ_min_ = −0.24 e Å^−3^

### 

Data collection: *SMART* (Bruker, 2001 ▶); cell refinement: *SMART*; data reduction: *SAINT-Plus* (Bruker, 2001 ▶); program(s) used to solve structure: *SHELXS97* (Sheldrick, 2008 ▶); program(s) used to refine structure: *SHELXL97* (Sheldrick, 2008 ▶); molecular graphics: *PLATON* (Spek, 2003 ▶); software used to prepare material for publication: *PLATON*.

## Supplementary Material

Crystal structure: contains datablocks global, I. DOI: 10.1107/S1600536808018369/at2577sup1.cif

Structure factors: contains datablocks I. DOI: 10.1107/S1600536808018369/at2577Isup2.hkl

Additional supplementary materials:  crystallographic information; 3D view; checkCIF report

## Figures and Tables

**Fig. 1.**
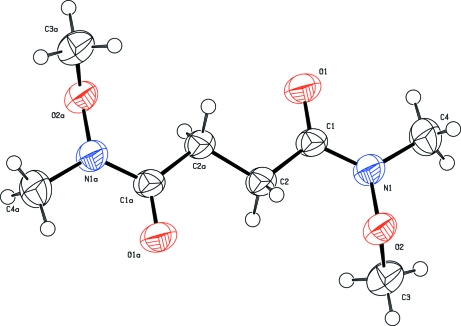
The molecular structure of (I), showing the atom-labelling scheme. Displacement ellipsoids are drawn at the 50% probability level.

## supplementary crystallographic information

### Comment

The Weinreb amides are widely recognized as effective acylating agents since they react with organometallics (RM, *M* = MgBr, Li) to produce ketones without side products in organic synthesis, including the total synthesis of complex natural products (Nahm & Weinreb, 1981). We here reported the structure of the Weinreb amides related title compound, (I).

Compound (I), is the synthetic intermediate, whose molecule is the centrosymmetric structure (Fig.1). In the symmetric unit, the C1—O1 bond distance is 1.224 (2) Å, which displays a typical double-bond of ketone carbonyl. Whereas, the N1—C1 bond distance of 1.342 (2) Å is obviously shorter than N1—C4 of 1.445 (2) Å, indicates that amide bond N1—C1 has some proporties of double-bond.

### Experimental

Triethylamine (25 ml, 180 mmol) was added slowly by cannulation to a stirred suspension of *N*,*O*-dimethylhydroxylamine (9.0 g, 92.25 mmol) and succinyl chloride (100 ml) in dichloromethane at 273 K under N_2_. After stirring for 2 h the solution was allowed to warm to room temperature and quenched with saturated aqueous sodium bicarbonate solution (50 ml). The layers were separated and the aqueous layer was extracted with dichloromethane (2×25 ml). The combined organic extracts were washed with brine (18.5 ml), dried (MgSO_4_) and evaporated under reduced pressure to give the compound (I) (7.365 g, 83%) as light brown needles. The molecule formula, C_8_H_16_N_2_O_4_ was established by EIMS m/*z*:144(*M*+ –N(CH_3_)OCH_3_). Spectroscopic analysis, ^1^H NMR (400 MHz; CDCl_3_-d_6_) δ:3.75 (6*H*, s, OCH_3_), 3.19 (6*H*, s, NCH_3_) and 2.78 (4*H*, s, CH_2_); ^13^C NMR (400 MHz; CDCl_3_-d_6_) δ:173.8 (C=O), 61.6 (OCH_3_), 32.6 and 26.8.

### Refinement

H atoms were treated as riding, with C—H distances in the range of 0.96–0.97 Å, and were refined as riding with *U*_iso_(*H*) =1.2*U*_eq_(C_methylene_) and *U*_iso_(*H*)=1.5*U*_eq_(C_methyl_).

### Crystal data

**Table:**

C_8_H_16_N_2_O_4_	*F*_000_ = 220
*M**_r_* = 204.23	*D*_x_ = 1.291 Mg m^−^^3^
Monoclinic, *P*2_1_/*c*	Mo *K*α radiation λ = 0.71073 Å
Hall symbol: -P 2ybc	Cell parameters from 925 reflections
*a* = 4.2645 (15) Å	θ = 2.6–26.6º
*b* = 11.152 (4) Å	µ = 0.10 mm^−^^1^
*c* = 11.165 (4) Å	*T* = 296 (2) K
β = 98.485 (5)º	Block, yellow
*V* = 525.2 (3) Å^3^	0.20 × 0.16 × 0.13 mm
*Z* = 2	

### Data collection

**Table:**

Bruker SMART APEX CCD area-detector diffractometer	909 independent reflections
Radiation source: fine-focus sealed tube	776 reflections with *I* > 2σ(*I*)
Monochromator: graphite	*R*_int_ = 0.015
*T* = 296(2) K	θ_max_ = 25.0º
φ and ω scans	θ_min_ = 2.6º
Absorption correction: multi-scan(SADABS; Sheldrick, 2001)	*h* = −5→5
*T*_min_ = 0.980, *T*_max_ = 0.987	*k* = −9→13
2116 measured reflections	*l* = −13→11

### Refinement

**Table:**

Refinement on *F*^2^	Secondary atom site location: difference Fourier map
Least-squares matrix: full	Hydrogen site location: inferred from neighbouring sites
*R*[*F*^2^ > 2σ(*F*^2^)] = 0.055	H-atom parameters constrained
*wR*(*F*^2^) = 0.174	*w* = 1/[σ^2^(*F*_o_^2^) + (0.117*P*)^2^ + 0.1547*P*] where *P* = (*F*_o_^2^ + 2*F*_c_^2^)/3
*S* = 1.01	(Δ/σ)_max_ < 0.001
909 reflections	Δρ_max_ = 0.25 e Å^−^^3^
64 parameters	Δρ_min_ = −0.24 e Å^−^^3^
Primary atom site location: structure-invariant direct methods	Extinction correction: none

### Special details

**Table:**

Geometry. All e.s.d.'s (except the e.s.d. in the dihedral angle between two l.s. planes) are estimated using the full covariance matrix. The cell e.s.d.'s are taken into account individually in the estimation of e.s.d.'s in distances, angles and torsion angles; correlations between e.s.d.'s in cell parameters are only used when they are defined by crystal symmetry. An approximate (isotropic) treatment of cell e.s.d.'s is used for estimating e.s.d.'s involving l.s. planes.
Refinement. Refinement of *F*^2^ against ALL reflections. The weighted *R*-factor *wR* and goodness of fit *S* are based on *F*^2^, conventional *R*-factors *R* are based on *F*, with *F* set to zero for negative *F*^2^. The threshold expression of *F*^2^ > σ(*F*^2^) is used only for calculating *R*-factors(gt) *etc*. and is not relevant to the choice of reflections for refinement. *R*-factors based on *F*^2^ are statistically about twice as large as those based on *F*, and *R*- factors based on ALL data will be even larger.

### Fractional atomic coordinates and isotropic or equivalent isotropic displacement parameters (Å^2^)

**Table:**

	*x*	*y*	*z*	*U*_iso_*/*U*_eq_	
O1	0.2198 (3)	0.51856 (11)	0.21773 (11)	0.0633 (4)	
O2	0.4066 (3)	0.76550 (10)	0.05213 (10)	0.0498 (3)	
N1	0.3748 (4)	0.69395 (13)	0.15260 (12)	0.0525 (4)	
C1	0.2269 (4)	0.58794 (14)	0.13325 (15)	0.0434 (4)	
C2	0.0721 (4)	0.56191 (14)	0.00580 (15)	0.0452 (4)	
H2A	0.2289	0.5689	−0.0485	0.054*	
H2B	−0.0920	0.6210	−0.0183	0.054*	
C3	0.2116 (5)	0.87038 (16)	0.0512 (2)	0.0643 (6)	
H3A	0.2351	0.9186	−0.0181	0.096*	
H3B	0.2754	0.9159	0.1237	0.096*	
H3C	−0.0061	0.8468	0.0475	0.096*	
C4	0.5720 (5)	0.72712 (18)	0.26424 (17)	0.0616 (5)	
H4A	0.5347	0.6728	0.3274	0.092*	
H4B	0.5214	0.8073	0.2861	0.092*	
H4C	0.7910	0.7232	0.2535	0.092*	

### Atomic displacement parameters (Å^2^)

**Table:**

	*U*^11^	*U*^22^	*U*^33^	*U*^12^	*U*^13^	*U*^23^
O1	0.0974 (10)	0.0429 (6)	0.0457 (7)	−0.0074 (6)	−0.0023 (6)	0.0066 (5)
O2	0.0637 (7)	0.0412 (6)	0.0458 (7)	−0.0059 (5)	0.0127 (5)	0.0014 (5)
N1	0.0754 (9)	0.0421 (7)	0.0376 (8)	−0.0108 (7)	0.0004 (7)	−0.0014 (6)
C1	0.0550 (9)	0.0329 (7)	0.0413 (9)	0.0029 (6)	0.0035 (7)	0.0003 (7)
C2	0.0568 (9)	0.0341 (8)	0.0425 (9)	−0.0008 (7)	0.0003 (7)	−0.0004 (7)
C3	0.0740 (12)	0.0423 (10)	0.0760 (13)	−0.0006 (8)	0.0094 (10)	0.0030 (9)
C4	0.0767 (12)	0.0605 (11)	0.0445 (10)	−0.0137 (9)	−0.0015 (9)	−0.0093 (9)

### Geometric parameters (Å, °)

**Table:**

O1—C1	1.2238 (19)	C2—H2B	0.9700
O2—N1	1.3994 (18)	C3—H3A	0.9600
O2—C3	1.434 (2)	C3—H3B	0.9600
N1—C1	1.342 (2)	C3—H3C	0.9600
N1—C4	1.445 (2)	C4—H4A	0.9600
C1—C2	1.506 (2)	C4—H4B	0.9600
C2—C2^i^	1.510 (3)	C4—H4C	0.9600
C2—H2A	0.9700		
			
N1—O2—C3	110.25 (13)	O2—C3—H3A	109.5
C1—N1—O2	118.16 (13)	O2—C3—H3B	109.5
C1—N1—C4	124.34 (15)	H3A—C3—H3B	109.5
O2—N1—C4	115.63 (14)	O2—C3—H3C	109.5
O1—C1—N1	119.82 (15)	H3A—C3—H3C	109.5
O1—C1—C2	123.31 (15)	H3B—C3—H3C	109.5
N1—C1—C2	116.87 (14)	N1—C4—H4A	109.5
C1—C2—C2^i^	111.95 (17)	N1—C4—H4B	109.5
C1—C2—H2A	109.2	H4A—C4—H4B	109.5
C2^i^—C2—H2A	109.2	N1—C4—H4C	109.5
C1—C2—H2B	109.2	H4A—C4—H4C	109.5
C2^i^—C2—H2B	109.2	H4B—C4—H4C	109.5
H2A—C2—H2B	107.9		
			
C3—O2—N1—C1	110.95 (17)	O2—N1—C1—C2	−7.3 (2)
C3—O2—N1—C4	−83.94 (19)	C4—N1—C1—C2	−171.05 (17)
O2—N1—C1—O1	173.52 (15)	O1—C1—C2—C2^i^	−3.6 (3)
C4—N1—C1—O1	9.8 (3)	N1—C1—C2—C2^i^	177.24 (18)

Symmetry codes: (i) −*x*, −*y*+1, −*z*.

## References

[bb1] Bruker (2001). *SAINT-Plus* and *SMART* Bruker AXS Inc., Madison, Wisconsin, USA.

[bb2] Nahm, S. & Weinreb, S. M. (1981). *Tetrahedron Lett.* **22**, 3815–3818.

[bb3] Sheldrick, G. M. (2001). *SADABS* Bruker AXS Inc., Madison, Wisconsin, USA.

[bb4] Sheldrick, G. M. (2008). *Acta Cryst.* A**64**, 112–122. 10.1107/S0108767307043930 18156677

[bb5] Spek, A. L. (2003). *J. Appl. Cryst.* **36**, 7–13.

